# Mediterranean diet adherence, social media use, and anxiety symptoms: an exploratory cross-sectional pilot survey

**DOI:** 10.3389/fnut.2026.1883903

**Published:** 2026-10-14

**Authors:** Piotr Religa, Michel-Edwar Mickael, Norwin Kubick, Inmaculada Conesa Moreno, Daniel Ferris Garcia, Mariusz Sacharczuk, Atanas G. Atanasov, Jarosław Olav Horbańczuk

**Affiliations:** 1 Department of Medicine, Solna, Karolinska Institutet, Stockholm, Sweden; 2 Institute of Genetics and Animal Biotechnology, Polish Academy of Sciences, Jastrzębiec, Poland; 3 Universität Hamburg, Hamburg, Germany; 4 Universidad de Murcia, Murcia, Spain; 5 Universidade de Santiago de Compostela, Santiago de Compostela, Spain

**Keywords:** anxiety, cross-sectional study, depression, HADS, Mediterranean diet, social media

## Abstract

**Background:**

Anxiety and depressive symptoms are common, and dietary patterns have been proposed as a modifiable influence on them. The majority of the evidence rests on composite adherence indices such as the Mediterranean Diet Adherence Screener (MEDAS), which sums its components as if they all contributed equally and addresses depression more often than anxiety. Whether individual dietary components relate to symptoms independently of the composite index has rarely been tested, and rarely alongside social media use, a further exposure proposed to shape both diet and mood.

**Methods:**

An anonymous online survey in English, Spanish, and Russian was completed by 115 adults aged 18 to 65 years with no previous diagnosis of mental illness, comprising a 14-item modified Mediterranean Diet Score (mMEDS), the Hospital Anxiety and Depression Scale (HADS), and questions on daily time spent on Instagram, Facebook, and Twitter/X. Four primary hypotheses tested the associations of olive oil intake and red and processed meat intake with each HADS subscale; all other comparisons were exploratory. The Benjamini–Hochberg correction was applied within each family. Effect sizes are reported with bootstrap 95% confidence intervals. The primary comparisons were additionally adjusted for sex, age band, and educational level.

**Results:**

All four primary estimates lay in the hypothesized direction. Higher olive oil intake was associated with lower anxiety scores (*r* = −0.249, 95% CI − 0.448 to −0.050, *p* = 0.021), and restricted red and processed meat intake was associated with lower anxiety and depression scores after adjustment (*β* = −2.12, *p* = 0.021; *β* = −1.62, *p* = 0.032). None survived correction for multiple testing. The mMEDS total did not appear to be related to either subscale (*ρ* = −0.045 and −0.141), while item-level associations differed in direction across food groups.

**Conclusion:**

Component-level signals appeared where the composite showed none, which may indicate that summing adherence items can obscure relationships running in opposite directions between foods. For studies using MEDAS-type indices, testing components alongside the total score could be worthwhile. Olive oil quantity and red and processed meat intake may be candidates for confirmatory work.

## Introduction

Anxiety disorders and major depressive disorders collectively affect an estimated 970 million individuals worldwide (1). Together, they represent one of the most prevalent forms of mental illness (2). Personal consequences of suffering from anxiety or depression include impaired occupational functioning, relationship difficulties, an elevated risk of physical comorbidity, and premature mortality (3). Both conditions follow a relapsing–remitting course in a substantial proportion of patients (4). Overall, 68% of patients with depression do not reach remission after a first-line course of antidepressant treatment. One-third of individuals with depression fail to achieve an adequate response to first-line pharmacological treatments (5, 6). Depression ranks as the fourth leading cause of the global burden of disease (7). Anxiety disorders impose comparable disability-adjusted costs (8).

Diet is increasingly recognized as an important factor influencing mental health (9). The growing burden of anxiety and depression has increased interest in lifestyle factors that may improve mental health (10). However, the association between adherence to healthy diet patterns, such as the Mediterranean diet, and anxiety and depression has been paradoxical. Several research groups have reported an association between adherence to Mediterranean-style dietary patterns and lower rates of depression and anxiety (11, 12). However, these results are not always consistent, with other studies showing weak or indirectly mediated associations (13, 14).

The conflicting findings reported in previous studies may be related to variability across social, ethnic, educational, sex, and age groups, as well as differences in physical activity and other lifestyle factors. One potentially important factor that is often overlooked is social media use (15). Meta-analyses have shown that greater problematic social media use is associated with higher levels of anxiety and depression, particularly among younger adults (16–18). However, whether extensive social media use may shape individual food choices, and hence anxiety or depression, is still unknown.

To address these limitations, we conducted a cross-sectional pilot study using an international online survey assessing individual Mediterranean diet component adherence using the validated 14-item PREDIMED instrument; anxiety and depression using the Hospital Anxiety and Depression Scale; and social media use across Instagram, Facebook, and Twitter/X. We applied several statistical methods to try to untangle the complex network of dietary and social media choices that might be correlated with anxiety and depression.

## Methods

### Study design and participants

Data were collected through an anonymous, cross-sectional online survey administered between 30 December 2023 and 25 January 2024 using Google Forms. Eligible participants were adults aged 18 to 65 years old without a previous diagnosis of mental illness. Recruitment was by convenience sampling: the authors and their students distributed the survey link through personal, institutional, and social media networks. The survey was conducted in English, Spanish, and Russian. The instruments were taken from their published English versions, and the Spanish and Russian versions were translated from these by native speakers of each language. Responses were harmonized to a single English label set before scoring. All three language versions are provided in Supplementary Material S1.

The country or region of residence was not recorded in the questionnaire. Since recruitment used open distribution of a survey link, the number of individuals who viewed the invitation is unknown, and a response rate cannot be calculated. No personal identifiers or IP addresses were collected, so no formal duplicate-prevention mechanism was applied. All 115 responses received were retained; no participant was excluded from the study. Where a participant did not answer an individual item, that participant was excluded pairwise from the comparisons involving that item only, and the number of participants contributing to each comparison is reported in the corresponding table. Both HADS subscales were complete for all 115 participants. Social media usage was reported by 98 participants for Instagram, 98 for Facebook, and 97 for Twitter/X. Participant flow is shown in Figure 1.

**Figure 1:**
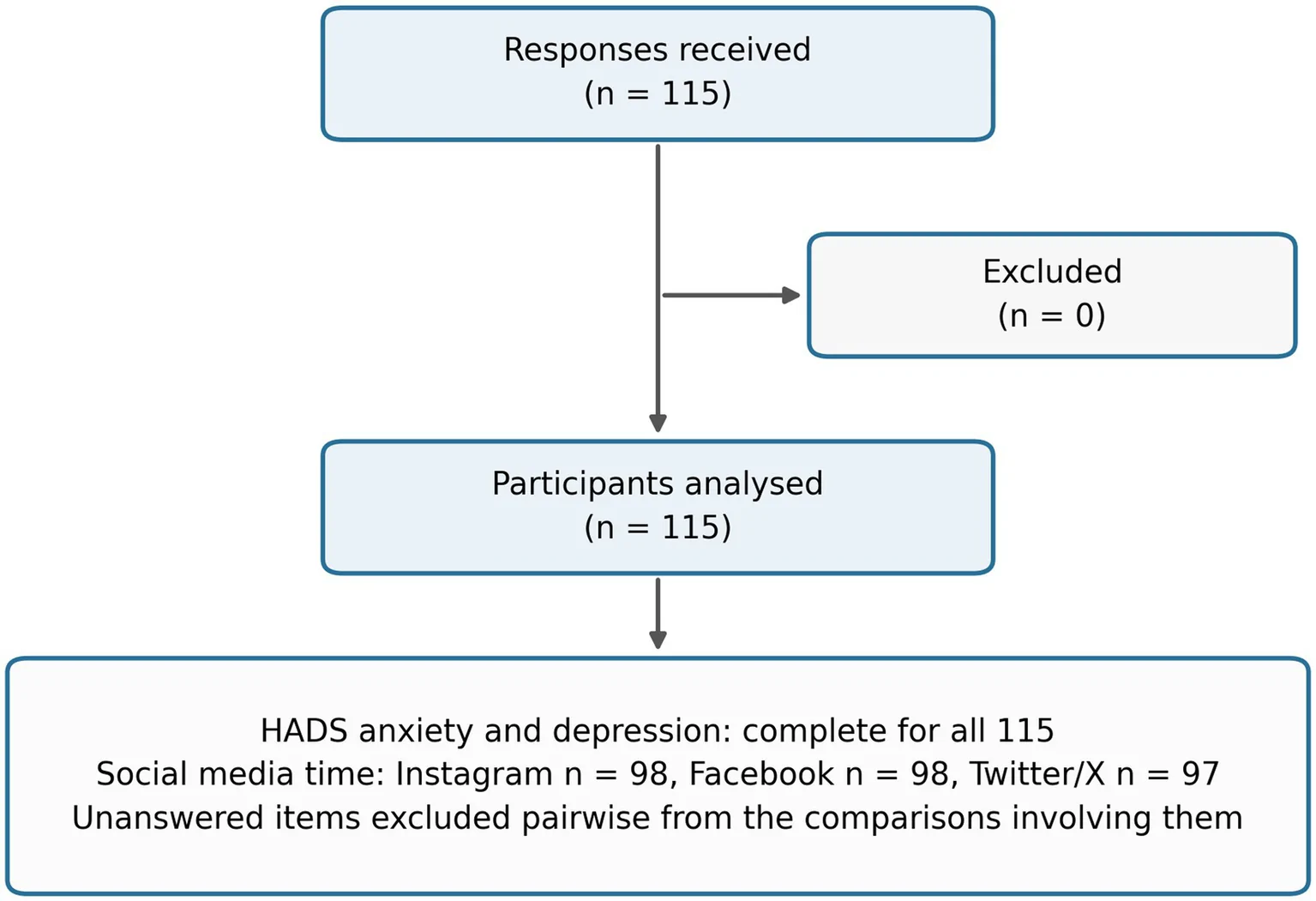
Participant flow. Responses received (*n* = 115); excluded (*n* = 0); participants analyzed (*n* = 115). Individual unanswered items were excluded pairwise from the comparisons involving them. Both HADS subscales were complete for all 115 participants. Social media use was reported by 98 participants for Instagram, 98 for Facebook, and 97 for Twitter/X.

This was a pilot study using a convenience sample, and the final sample size of 115 participants reflects the number of respondents obtained during the recruitment window. No *a priori* sample size calculation was performed, and the analyses are accordingly presented as exploratory and hypothesis-generating. Precision is conveyed by reporting a confidence interval for every estimate.

### Ethics

The study was approved by the, Institute of Scientific Personnel Training of the National Academy of Sciences. Approval was granted in August 2021 for the research program under which this survey was conducted. All participants provided informed consent before beginning the survey by signing an informed consent sheet presented at the start of the questionnaire. No personal identifiers or IP addresses were collected, and data handling conformed to the General Data Protection Regulation (GDPR) requirements.

### Dietary assessment

Diet was assessed using a modified 14-item Mediterranean Diet Score (mMEDS), adapted from the PREDIMED Mediterranean Diet Adherence Screener (MEDAS) (19, 20). The items cover the following: olive oil as the main cooking fat; daily olive oil consumption of four or more tablespoons; homemade vegetable sauces at least twice weekly; four or more daily servings of vegetables; three or more daily servings of fruit; nuts at least three times weekly; fish at least three times weekly; legumes at least three times weekly; preference for poultry over red meat; fewer than two weekly servings of red or processed meat; less than one daily tablespoon of butter, margarine, or cream; two or fewer weekly servings of processed snacks; fewer than one daily can of sugary beverages; and consumption of more than seven glasses of wine per week. Each item was scored 1 for adherence and 0 for non-adherence, giving a total score range of 0 to 14. No item was reverse-coded. Three items were administered at thresholds specific to this survey: vegetable intake of four or more servings daily, red and processed meat intake of fewer than two servings weekly, and wine intake of more than seven glasses weekly. The remaining 11 items applied the original criteria. This composite is referred to throughout as the mMEDS and is not compared with published MEDAS distributions (Figure 2).

**Figure 2:**
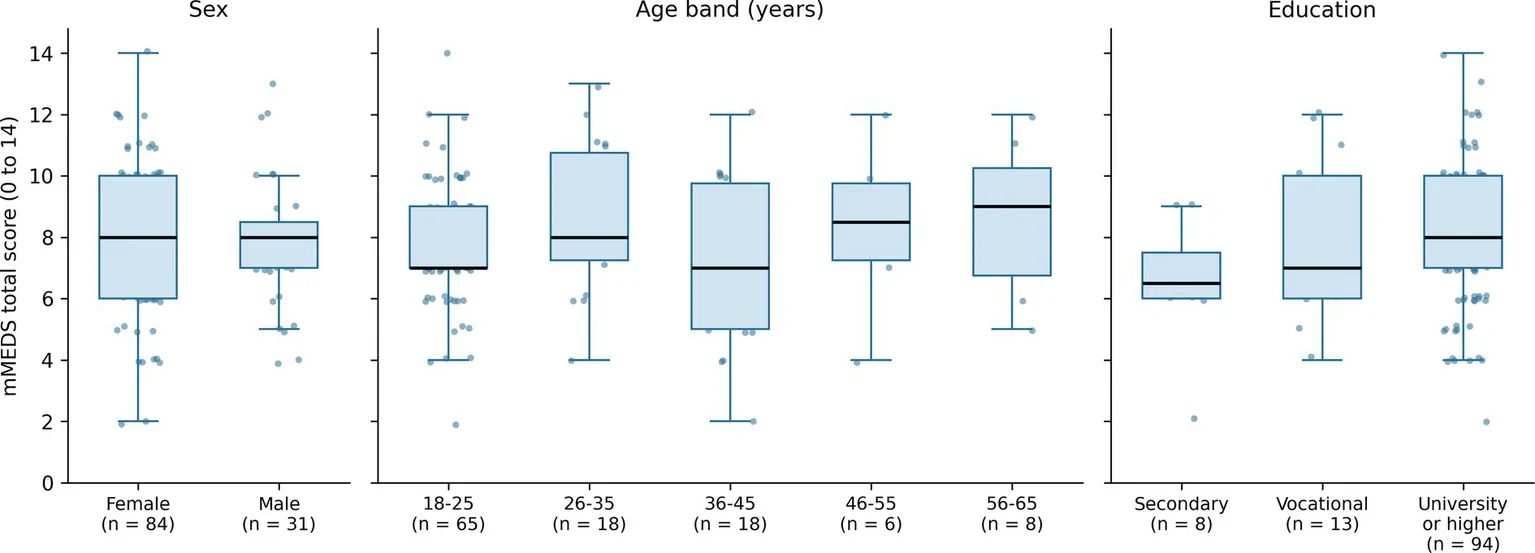
Distribution of mMEDS total scores across demographic groups.

### Psychological assessment

Anxiety and depressive symptoms were assessed using the Hospital Anxiety and Depression Scale (HADS) (21), a 14-item instrument comprising a 7-item anxiety subscale and a 7-item depression subscale, each item scored 0 to 3 and each subscale ranging from 0 to 21. Scores were classified as normal (0–7), borderline (8–10), or abnormal (11–21). Internal consistency in this sample was acceptable for both subscales (Cronbach’s α = 0.83, 95% CI 0.77 to 0.87 for anxiety; α = 0.72, 95% CI 0.59 to 0.81 for depression, bootstrap intervals from 5,000 resamples) (Figure 3).

**Figure 3:**
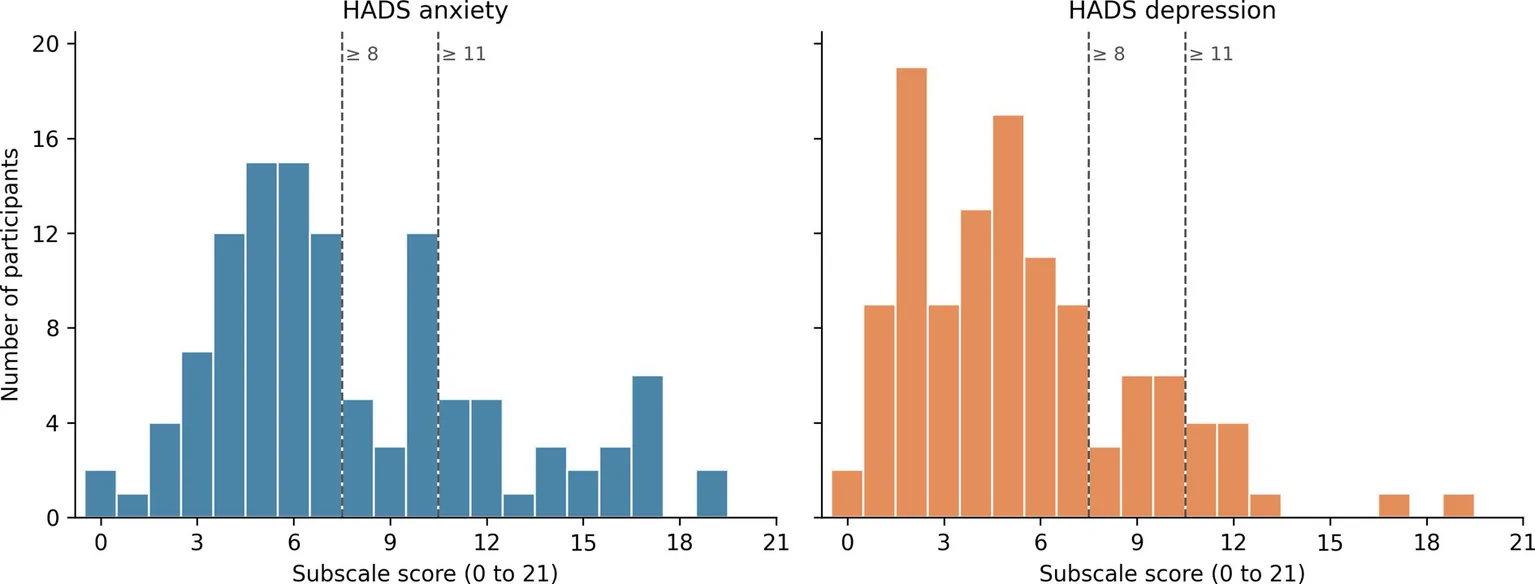
Distribution of HADS anxiety and depression scores.

### Social media assessment

Participants first indicated whether they used social media. Those who responded were asked to estimate their average daily time spent on Instagram, Facebook, and Twitter/X by selecting one of the five fixed response categories: 10 min or less, 30 min, 50 min, 70 min, and 90 min or more. These were pre-specified categories rather than free-text estimates, and they were assigned values of 10, 30, 50, 70, and 90 min for the analysis. The scale was censored at both ends, and no zero-min option was offered, so non-use of an individual platform was not separately identifiable. Only Instagram, Facebook, and Twitter/X were assessed (Figure 4).

**Figure 4:**
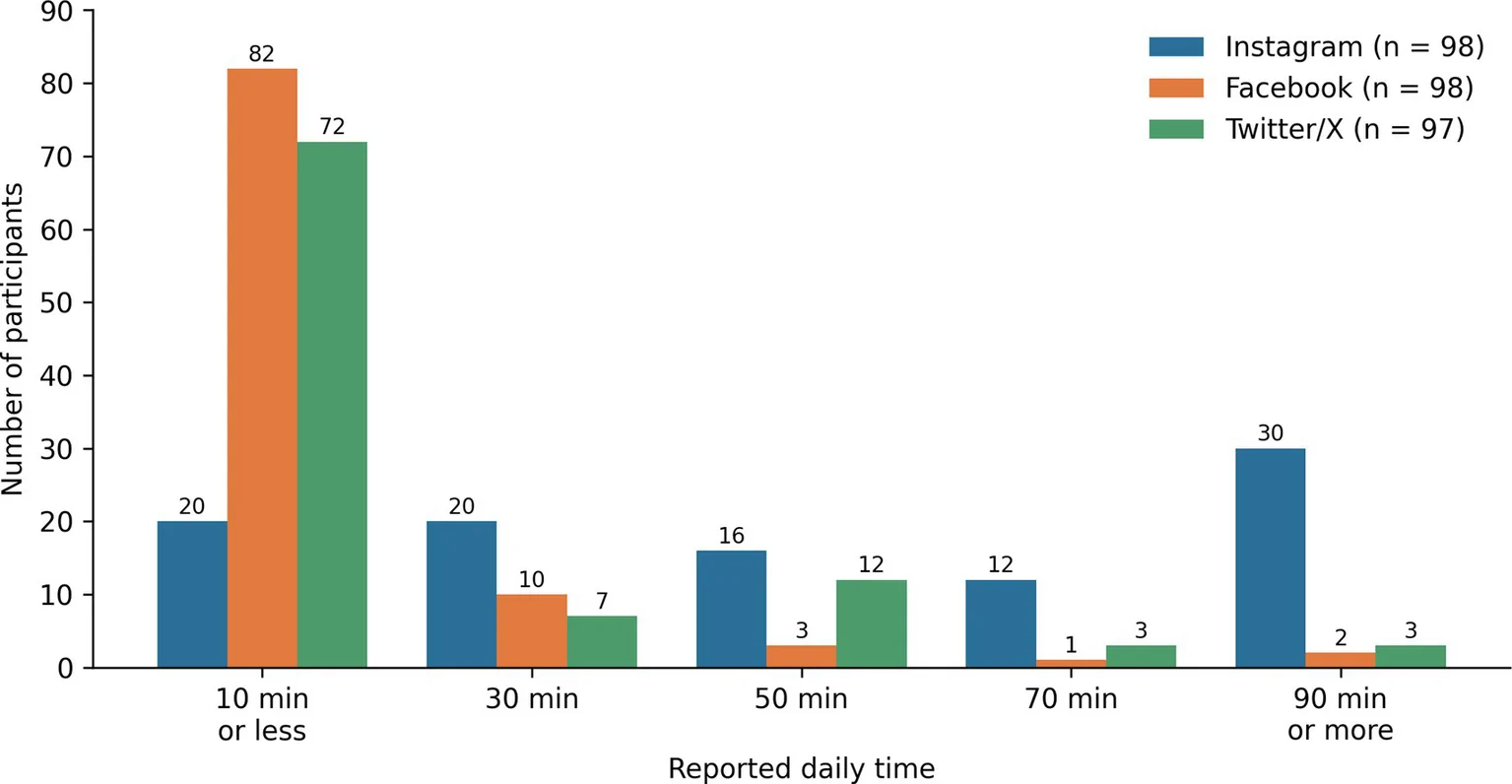
Distribution of reported daily social media use by platform. Grouped bar chart showing the number of participants in each of the five daily time categories (10 min or less, 30, 50, 70, 90, or more) for Instagram, Facebook, and Twitter/X.

### Hypotheses

Four primary hypotheses were specified, covering two dietary components for which prior evidence exists, each tested with both HADS subscales. The evidence base addresses both anxiety and depressive symptoms, and hypotheses were specified for both outcomes accordingly:

*H1*. Daily consumption of four or more tablespoons of olive oil is associated with lower HADS anxiety scores.

*H2*. Daily consumption of four or more tablespoons of olive oil is associated with lower HADS depression scores.

*H3*. Consumption of fewer than two weekly servings of red or processed meat is associated with lower HADS anxiety scores.

*H4*. Consumption of fewer than two weekly servings of red or processed meat is associated with lower HADS depression scores.

### Statistical analysis

Continuous scores were compared between the adherent and non-adherent groups using the Mann–Whitney U test, with group-specific sample sizes, medians, and interquartile ranges reported alongside the U statistic. Effect size is expressed as the rank–biserial correlation, calculated as *r* = 2 U/(*n*₁*n*₂) – 1, with U computed for the adherent group, so that a negative value indicates lower outcome scores among adherent participants. Confidence intervals were obtained by bootstrap resampling within the groups (5,000 resamples). Comparisons across three or more groups used the Kruskal–Wallis test and were reported with the H statistic, degrees of freedom, and epsilon-squared. Associations between continuous measures used Spearman’s rank correlation. Associations between social media time and the binary adherence items were tested using the Mann–Whitney U test, comparing the distribution of reported daily minutes between adherent and non-adherent participants. As rank-based methods depend only on the ordering of the response categories, the specific minute values assigned do not affect the results. One hypothesis family was defined per outcome variable, and the Benjamini–Hochberg procedure was applied to control the false discovery rate within each family, with both unadjusted and adjusted values reported for every comparison. For the four primary comparisons, multivariable linear regression models were fitted with the HADS subscale score as the dependent variable and the dietary item, sex, age band, and educational level as independent variables. Analyses were conducted in Python 3 using pandas, SciPy, and statsmodels. Missing responses were handled by pairwise exclusion.

## Results

### Sample characteristics

Of the 115 participants, 84 (73.0%) were women, and 31 (27.0%) were men. Sixty-five participants (56.5%) were aged 18–25 years, with the remaining distributed across the 26–35 (*n* = 18), 36–45 (*n* = 18), 46–55 (*n* = 6), and 56–65 (*n* = 8) bands. Ninety-four participants (81.7%) held a university degree or higher, 13 (11.3%) had completed vocational training, and 8 (7.0%) had completed secondary studies.

The mMEDS total score had a mean of 7.88 (SD 2.33), a median of 8 (IQR 6–10), and a range of 2–14. HADS anxiety had a mean of 7.84 (SD 4.41), a median of 7 (IQR 5–10), and a range of 0–19; HADS depression had a mean of 5.41 (SD 3.62), a median of 5 (IQR 2–7), and a range of 0–19. Sixty-eight participants (59.1%) fell in the normal range for anxiety, 20 (17.4%) in the borderline range, and 27 (23.5%) in the abnormal range; for depression, the corresponding figures were 89 (77.4%), 15 (13.0%), and 11 (9.6%). Both subscales were complete for all 115 participants (Table 1).

**Table 1:** Characteristics of the study sample (*n* = 115).

Characteristic	Level	*n*	%
Sex	Female	84	73.0
Sex	Male	31	27.0
Age band	18–25	65	56.5
Age band	26–35	18	15.7
Age band	36–45	18	15.7
Age band	46–55	6	5.2
Age band	56–65	8	7.0
Education	University degree or higher	94	81.7
Education	Vocational training module	13	11.3
Education	Secondary studies	8	7.0
HADS anxiety category	Normal (0–7)	68	59.1
HADS anxiety category	Borderline (8–10)	20	17.4
HADS anxiety category	Abnormal (11–21)	27	23.5
HADS depression category	Normal (0–7)	89	77.4
HADS depression category	Borderline (8–10)	15	13.0
HADS depression category	Abnormal (11–21)	11	9.6

Distribution of scores				
Measure	*n*	Mean (SD)	Median (IQR)	Range
mMEDS total score (0–14)	115	7.88 (2.33)	8 (6–10)	2–14
11-item score (0–11)	115	6.72 (1.94)	7 (6–8)	1–11
HADS anxiety (0–21)	115	7.84 (4.41)	7 (5–10)	0–19
HADS depression (0–21)	115	5.41 (3.62)	5 (2–7)	0–19

mMEDS, 14-item modified Mediterranean Diet Score. HADS, Hospital Anxiety and Depression Scale. This 11-item score comprises the items administered at the original thresholds. Percentages are of the full analytic sample (*n* = 115).

### Primary comparisons

All four primary comparisons were in the hypothesized direction, and none remained significant after correction (Table 2).

**Table 2:** Mediterranean diet items and HADS subscale scores.

Item	Outcome	*n* adherent	*n* non-adherent	Median (IQR) adherent	Median (IQR) non-adherent	U	p	*r*	95% CI	*q*
Primary comparisons (Benjamini–Hochberg correction applied across the four primary tests).										
Olive oil, ≥4 tablespoons/day	HADS anxiety	59	56	6 (4–10)	8 (6–11)	1241.0	0.0211	−0.249	[−0.448, −0.050]	0.084
Olive oil, ≥4 tablespoons/day	HADS depression	59	56	5 (2–7)	5 (3–7)	1484.0	0.3458	−0.102	[−0.307, 0.109]	0.346
Red or processed meat, <2 servings/week	HADS anxiety	79	36	6 (4–10)	9 (5–12)	1112.0	0.0608	−0.218	[−0.443, 0.019]	0.110
Red or processed meat, <2 servings/week	HADS depression	79	36	5 (2–7)	6 (4–7)	1135.5	0.0827	−0.201	[−0.412, 0.015]	0.110
Exploratory comparisons (Benjamini–Hochberg correction applied within each outcome, 12 tests per outcome).										
Olive oil as main cooking fat	HADS anxiety	99	16	7 (4–10)	8 (6–11)	646.5	0.2393	−0.184	[−0.472, 0.120]	0.949
Homemade vegetable sauces, ≥2/week	HADS anxiety	65	50	7 (5–11)	7 (5–10)	1741.5	0.5110	+0.072	[−0.138, 0.281]	0.949
Vegetables, ≥4 servings/day	HADS anxiety	43	72	7 (5–10)	6 (5–11)	1568.5	0.9076	+0.013	[−0.199, 0.227]	0.949
Fruit, ≥3 servings/day	HADS anxiety	51	64	8 (4–10)	6 (5–10)	1752.0	0.4993	+0.074	[−0.144, 0.290]	0.949
Nuts, ≥3 times/week	HADS anxiety	66	49	6 (5–10)	7 (5–11)	1449.5	0.3428	−0.104	[−0.313, 0.112]	0.949
Fish, ≥3 times/week	HADS anxiety	43	72	7 (5–10)	6 (5–11)	1595.0	0.7872	+0.030	[−0.191, 0.251]	0.949
Legumes, ≥3 times/week	HADS anxiety	50	65	7 (5–10)	6 (5–11)	1666.5	0.8163	+0.026	[−0.187, 0.237]	0.949
Poultry preferred over red meat	HADS anxiety	99	16	7 (5–10)	6 (4–11)	830.5	0.7578	+0.049	[−0.306, 0.393]	0.949
Butter, margarine, or cream, <1 tablespoon/day	HADS anxiety	85	30	6 (5–10)	8 (6–10)	1116.5	0.3122	−0.124	[−0.358, 0.111]	0.949
Processed snacks, ≤2 servings/week	HADS anxiety	63	52	7 (4–10)	6 (5–10)	1611.5	0.8833	−0.016	[−0.226, 0.190]	0.949
Sugary beverages, <1 can/day	HADS anxiety	93	22	7 (5–10)	6 (5–10)	1032.5	0.9488	+0.009	[−0.242, 0.264]	0.949
Wine, >7 glasses/week	HADS anxiety	11	104	8 (6–16)	7 (5–10)	735.5	0.1196	+0.286	[−0.093, 0.661]	0.949
Olive oil as main cooking fat	HADS depression	99	16	5 (2–7)	5 (4–8)	728.0	0.6057	−0.081	[−0.364, 0.218]	0.982
Homemade vegetable sauces, ≥2/week	HADS depression	65	50	5 (2–7)	5 (3–7)	1567.5	0.7463	−0.035	[−0.244, 0.168]	0.982
Vegetables, ≥4 servings/day	HADS depression	43	72	4 (2–6)	5 (3–7)	1297.5	0.1461	−0.162	[−0.369, 0.045]	0.982
Fruit, ≥3 servings/day	HADS depression	51	64	4 (2–8)	5 (3–7)	1440.0	0.2782	−0.118	[−0.333, 0.105]	0.982
Nuts, ≥3 times/week	HADS depression	66	49	5 (2–7)	5 (3–8)	1423.0	0.2710	−0.120	[−0.321, 0.091]	0.982
Fish, ≥3 times/week	HADS depression	43	72	5 (2–9)	5 (3–7)	1674.0	0.4656	+0.081	[−0.142, 0.300]	0.982
Legumes, ≥3 times/week	HADS depression	50	65	5 (3–7)	5 (2–7)	1561.0	0.7186	−0.039	[−0.250, 0.165]	0.982
Poultry preferred over red meat	HADS depression	99	16	5 (3–7)	5 (2–7)	854.5	0.6143	+0.079	[−0.275, 0.423]	0.982
Butter, margarine, or cream, <1 tablespoon/day	HADS depression	85	30	5 (3–7)	5 (2–8)	1303.5	0.8576	+0.022	[−0.224, 0.270]	0.982
Processed snacks, ≥2 servings/week	HADS depression	63	52	5 (2–7)	5 (3–7)	1633.5	0.9820	−0.003	[−0.214, 0.210]	0.982
Sugary beverages, <1 can/day	HADS depression	93	22	5 (2–7)	5 (3–8)	964.5	0.6783	−0.057	[−0.313, 0.200]	0.982
Wine, >7 glasses/week	HADS depression	11	104	3 (2–10)	5 (3–7)	583.0	0.9200	+0.019	[−0.379, 0.422]	0.982

Adherent, participants meeting the item criterion; non-adherent, those not meeting it. U, Mann–Whitney U statistic computed for the adherent group. *p*, unadjusted two-sided *p*-value. *q*, Benjamini–Hochberg adjusted *p*-value. *r*, rank–biserial correlation, calculated as 2 U/(*n*₁*n*₂) − 1, where a negative value indicates lower scores in the adherent group; 95% CI obtained by bootstrap resampling within groups (5,000 resamples). IQR, interquartile range. *n* = 115 for all comparisons.

Participants adherent to the olive oil quantity item had a median HADS anxiety score of 6 (IQR 4–10) compared with a score of 8 (IQR 6–11) among non-adherent participants (*U* = 1241.0, *p* = 0.021, *q* = 0.084, *r* = −0.249, 95% CI − 0.448 to −0.050). The same item compared with depression had medians of 5 (IQR 2–7) and 5 (IQR 3–7), respectively (*p* = 0.346, *q* = 0.346, *r* = −0.102, 95% CI − 0.307 to 0.109).

Participants adherent to the red and processed meat item had a median anxiety score of 6 (IQR 4–10) compared with a score of 9 (IQR 5–12) (*p* = 0.061, *q* = 0.110, *r* = −0.218, 95% CI − 0.443 to 0.019) among non-adherent participants, and those adherent to that item had a median depression score of 5 (IQR 2–7) compared with a score of 6 (IQR 4–7) (*p* = 0.083, *q* = 0.110, *r* = −0.201) among non-adherent participants.

In multivariable models adjusted for sex, age band, and educational level, three of the four associations reached nominal significance: olive oil and anxiety (*β* = −1.86, 95% CI − 3.54 to −0.18, *p* = 0.031), red and processed meat and anxiety (*β* = −2.12, 95% CI − 3.91 to −0.32, *p* = 0.021), and red and processed meat and depression (*β* = −1.62, 95% CI − 3.09 to −0.14, *p* = 0.032). Olive oil and depression did not reach nominal significance (*β* = −0.57, 95% CI − 1.98 to 0.83, *p* = 0.422). All models explained less than 10% of the variance in the outcome (*R*^2^ 0.054 to 0.095).

Overall adherence, tested as a single summary exposure, was not associated with either subscale (mMEDS with anxiety *ρ* = −0.045, 95% CI − 0.239 to 0.151, *p* = 0.63; and depression *ρ* = −0.141, 95% CI − 0.316 to 0.046, *p* = 0.13). An 11-item score restricted to items administered as originally specified gave the same results (*ρ* = −0.047, *p* = 0.62 and *ρ* = −0.097, *p* = 0.30, respectively), as did a 13-item score excluding only the vegetable item (*ρ* = −0.066, *p* = 0.48 and *ρ* = −0.126, *p* = 0.18).

### Exploratory comparisons: dietary items and psychological symptoms

Across the 12 remaining dietary items tested against each HADS subscale, no comparison reached *p* < 0.05 before correction; the smallest unadjusted values were 0.120 for anxiety and 0.146 for depression (Table 2).

Among demographic comparisons, women reported higher anxiety scores than men (*U* = 899.0, *p* = 0.011, *r* = −0.310), with no corresponding difference in depression (*p* = 0.466). Neither age band nor educational level was associated with either subscale (age band and anxiety H(4) = 0.355, *p* = 0.986; age band and depression H(4) = 2.098, *p* = 0.718; education and anxiety H(2) = 0.012, *p* = 0.994; education and depression H(2) = 2.222, *p* = 0.329; epsilon-squared below 0.01 throughout).

### Exploratory comparisons: dietary items and social media use

Seven of the 42 comparisons between dietary items and reported daily platform use reached *p*-values of less than 0.05 before correction, and one survived correction within its platform family (Table 3).

**Table 3:** Mediterranean diet items and daily social media use.

Item	Outcome	*n* adherent	*n* non-adherent	Median (IQR) adherent	Median (IQR) non-adherent	U	p	*r*	95% CI	*q*
Olive oil as main cooking fat	Instagram, min/day	83	15	50 (30–90)	30 (10–60)	823.5	0.0422	+0.323	[0.011, 0.616]	0.247
Olive oil, ≥4 tablespoons/day	Instagram, min/day	49	49	50 (30–90)	50 (30–90)	1272.5	0.6019	+0.060	[−0.173, 0.277]	0.861
Homemade vegetable sauces, ≥2/week	Instagram, min/day	59	39	50 (30–90)	50 (30–90)	1231.5	0.5485	+0.070	[−0.163, 0.289]	0.861
Vegetables, ≥4 servings/day	Instagram, min/day	35	63	30 (10–70)	50 (30–90)	752.5	0.0078	−0.317	[−0.537, −0.083]	0.109
Fruit, ≥3 servings/day	Instagram, min/day	45	53	30 (10–90)	50 (30–90)	959.5	0.0887	−0.195	[−0.414, 0.035]	0.310
Nuts, ≥3 times/week	Instagram, min/day	60	38	50 (30–70)	70 (30–90)	881.0	0.0529	−0.227	[−0.445, 0.011]	0.247
Fish, ≥3 times/week	Instagram, min/day	36	62	50 (30–90)	50 (30–90)	1091.0	0.8529	−0.022	[−0.255, 0.211]	0.908
Legumes, ≥3 times/week	Instagram, min/day	43	55	50 (30–90)	50 (30–90)	1277.5	0.4872	+0.080	[−0.138, 0.301]	0.861
Poultry preferred over red meat	Instagram, min/day	85	13	50 (30–90)	70 (30–90)	433.5	0.2025	−0.215	[−0.518, 0.115]	0.567
Red or processed meat, <2 servings/week	Instagram, min/day	64	34	50 (30–90)	50 (10–90)	1154.0	0.6156	+0.061	[−0.188, 0.299]	0.861
Butter, margarine, or cream, <1 tablespoon/day	Instagram, min/day	72	26	50 (30–90)	50 (30–85)	885.0	0.6765	−0.054	[−0.300, 0.184]	0.861
Processed snacks, ≤2 servings/week	Instagram, min/day	53	45	50 (30–90)	50 (10–90)	1262.5	0.6109	+0.059	[−0.170, 0.284]	0.861
Sugary beverages, <1 can/day	Instagram, min/day	79	19	50 (30–90)	50 (30–90)	737.5	0.9082	−0.017	[−0.300, 0.265]	0.908
Wine, >7 glasses/week	Instagram, min/day	10	88	40 (30–80)	50 (30–90)	425.0	0.8613	−0.034	[−0.372, 0.315]	0.908
Olive oil as main cooking fat	Facebook, min/day	83	15	10 (10–10)	10 (10–30)	450.0	0.0083	−0.277	[−0.533, −0.022]	0.058
Olive oil, ≥4 tablespoons/day	Facebook, min/day	49	49	10 (10–10)	10 (10–10)	1152.0	0.5957	−0.040	[−0.185, 0.104]	0.596
Homemade vegetable sauces, ≥2/week	Facebook, min/day	59	39	10 (10–10)	10 (10–10)	1314.5	0.0649	+0.143	[0.007, 0.274]	0.151
Vegetables, ≥4 servings/day	Facebook, min/day	35	63	10 (10–30)	10 (10–10)	1314.0	0.0149	+0.192	[0.031, 0.360]	0.070
Fruit, ≥3 servings/day	Facebook, min/day	45	53	10 (10–10)	10 (10–10)	1278.0	0.3458	+0.072	[−0.079, 0.220]	0.402
Nuts, ≥3 times/week	Facebook, min/day	60	38	10 (10–10)	10 (10–10)	1061.0	0.3732	−0.069	[−0.226, 0.085]	0.402
Fish, ≥3 times/week	Facebook, min/day	36	62	10 (10–30)	10 (10–10)	1319.5	0.0199	+0.182	[0.020, 0.357]	0.070
Legumes, ≥3 times/week	Facebook, min/day	43	55	10 (10–10)	10 (10–10)	1090.0	0.3055	−0.078	[−0.222, 0.070]	0.402
Poultry preferred over red meat	Facebook, min/day	85	13	10 (10–10)	10 (10–30)	466.0	0.1611	−0.157	[−0.435, 0.089]	0.282
Red or processed meat, <2 servings/week	Facebook, min/day	64	34	10 (10–10)	10 (10–25)	923.0	0.0561	−0.152	[−0.324, 0.015]	0.151
Butter, margarine, or cream, <1 tablespoon/day	Facebook, min/day	72	26	10 (10–10)	10 (10–25)	807.0	0.1077	−0.138	[−0.331, 0.047]	0.215
Processed snacks, ≤2 servings/week	Facebook, min/day	53	45	10 (10–10)	10 (10–10)	1107.0	0.3458	−0.072	[−0.222, 0.074]	0.402
Sugary beverages, <1 can/day	Facebook, min/day	79	19	10 (10–10)	10 (10–30)	518.0	0.0012	−0.310	[−0.546, −0.085]	0.017
Wine, >7 glasses/week	Facebook, min/day	10	88	10 (10–25)	10 (10–10)	504.5	0.2426	+0.147	[−0.125, 0.453]	0.377
Olive oil as main cooking fat	Twitter/X, min/day	82	15	10 (10–30)	10 (10–10)	753.5	0.0727	+0.225	[0.044, 0.378]	0.509
Olive oil, ≥4 tablespoons/day	Twitter/X, min/day	49	48	10 (10–30)	10 (10–10)	1249.5	0.4924	+0.062	[−0.109, 0.242]	0.739
Homemade vegetable sauces, ≥2/week	Twitter/X, min/day	58	39	10 (10–25)	10 (10–20)	1148.5	0.8705	+0.015	[−0.160, 0.195]	0.937
Vegetables, ≥4 servings/day	Twitter/X, min/day	34	63	10 (10–10)	10 (10–40)	823.0	0.0147	−0.232	[−0.381, −0.070]	0.206
Fruit, ≥3 servings/day	Twitter/X, min/day	45	52	10 (10–10)	10 (10–30)	1137.0	0.7593	−0.028	[−0.205, 0.141]	0.937
Nuts, ≥3 times/week	Twitter/X, min/day	59	38	10 (10–10)	10 (10–30)	1055.0	0.5281	−0.059	[−0.244, 0.126]	0.739
Fish, ≥3 times/week	Twitter/X, min/day	36	61	10 (10–10)	10 (10–30)	1031.5	0.5207	−0.061	[−0.231, 0.119]	0.739
Legumes, ≥3 times/week	Twitter/X, min/day	43	54	10 (10–20)	10 (10–25)	1157.5	0.9773	−0.003	[−0.177, 0.175]	0.977
Poultry preferred over red meat	Twitter/X, min/day	85	12	10 (10–10)	10 (10–50)	446.0	0.3646	−0.125	[−0.439, 0.169]	0.729
Red or processed meat, <2 servings/week	Twitter/X, min/day	63	34	10 (10–20)	10 (10–25)	1048.5	0.8284	−0.021	[−0.218, 0.159]	0.937
Butter, margarine, or cream, <1 tablespoon/day	Twitter/X, min/day	71	26	10 (10–30)	10 (10–10)	1063.5	0.1373	+0.152	[−0.029, 0.313]	0.638
Processed snacks, ≤2 servings/week	Twitter/X, min/day	52	45	10 (10–10)	10 (10–50)	1032.5	0.1965	−0.118	[−0.294, 0.058]	0.638
Sugary beverages, <1 can/day	Twitter/X, min/day	78	19	10 (10–30)	10 (10–10)	833.5	0.2758	+0.125	[−0.077, 0.308]	0.644
Wine, >7 glasses/week	Twitter/X, min/day	10	87	10 (10–50)	10 (10–10)	513.5	0.2278	+0.180	[−0.148, 0.521]	0.638

Daily use was reported in five fixed categories, which were assigned values of 10, 30, 50, 70, and 90 min; the scale was censored at both ends. Comparisons used the Mann–Whitney *U* test. *U*, Mann–Whitney *U* statistic computed for the adherent group. *p*, unadjusted two-sided *p*-value. *q*, Benjamini–Hochberg adjusted *p*-value, applied within each platform (14 tests per platform). *r*, rank–biserial correlation, where a positive value indicates greater reported use in the adherent group; 95% CI by bootstrap resampling within groups (5,000 resamples). IQR, interquartile range. Group sizes vary because participants who did not answer for a given platform were excluded pairwise (Instagram *n* = 98, Facebook *n* = 98, Twitter/X *n* = 97).

For Instagram, adherence to the vegetable item was associated with lower reported use (median 30 min, IQR 10–70, compared with 50 min, IQR 30–90; *p* = 0.008, *q* = 0.109, *r* = −0.317), whereas adherence to the olive oil cooking-fat item was associated with higher reported use (median 50 compared with 30 min; *p* = 0.042, *q* = 0.247, *r* = +0.323). No association was observed for the fruit item (*r* = −0.195, 95% CI −0.414 to 0.035, *p* = 0.089) or the legume item (*r* = +0.080, 95% CI −0.138 to 0.301, *p* = 0.487).

For Facebook, adherence to the sugary beverages item was associated with lower reported use (*p* = 0.001, *q* = 0.017, *r* = −0.310). This was the only association in the study to survive correction. Group medians were identical at 10 min, and the interquartile ranges differed only in the upper tail (10–10 compared with 10–30), indicating that the difference is confined to a minority of heavier users among non-adherent participants.

Overall adherence did not appear to be associated with the reported use of any platform (Instagram *ρ* = −0.060, *p* = 0.56; Facebook *ρ* = −0.059, *p* = 0.56; Twitter/X *ρ* = −0.038, *p* = 0.71), and neither HADS subscale appeared to be associated with platform use (Instagram–anxiety *ρ* = +0.023, *p* = 0.82; Facebook–anxiety *ρ* = +0.184, 95% CI 0.011 to 0.348, *p* = 0.070; Twitter/X–anxiety *ρ* = −0.097, *p* = 0.34).

## Discussion

In this exploratory pilot survey of 115 adults, all 4 primary point estimates lay in the hypothesized direction, and 3 of the 4 appeared to reach nominal significance once sex, age band, and educational level were accounted for, although none survived correction for multiple testing. The confidence intervals were found to be wide, and the adjusted models explained less than a tenth of the variance in symptom scores, so these associations may be compatible with the small effects described in larger studies rather than constituting independent evidence of them.

The results are reported for both subscales. Previous studies have examined the relation between dietary components and depressive symptoms (22–27): A prospective cohort of 12,059 Spanish graduates reported a weak inverse association between olive oil consumption and incident depression (22), a randomized trial in 73 patients with major depression reported symptom improvement following daily extra-virgin olive oil (23), and a meta-analysis of 17 studies covering 241,738 participants reported an association between red and processed meat intake and depression (25). In this study, restricted red and processed meat intake appeared to be associated with lower depression scores once sex, age band, and educational level were accounted for (*β* = −1.62, 95% CI − 3.09 to −0.14, *p* = 0.032), which could be consistent with that body of work, although the unadjusted comparison did not reach nominal significance (*p* = 0.083). Olive oil intake did not appear to be associated with depression scores (*p* = 0.346 unadjusted, *p* = 0.422 adjusted). Neither comparison survived correction for multiple testing, and the confidence intervals remained wide enough that effects of the magnitude reported in those larger studies cannot be excluded.

The closest published analog to our design is a randomized trial in adults with severe obesity that used the HADS as its outcome measure and reported reductions in both anxiety and depressive symptoms following daily extra-virgin olive oil (24). The direction of our olive oil estimates appears to match, while the magnitude does not, which might be expected: An intervention trial in a clinical population and a cross-sectional survey of a young, healthy, highly educated convenience sample are not comparable designs.

Associations between dietary items and social media use were found to be heterogeneous in direction. Adherence to the vegetable item appeared to be associated with lower reported Instagram use, while adherence to the olive oil cooking-fat item appeared to be associated with higher use, and overall adherence did not appear to be related to use of any platform. Reports in the literature are similarly mixed: Social media use has been associated with skipping breakfast, greater consumption of unhealthy snacks and sugar-sweetened beverages, and lower fruit and vegetable intake (28, 29), while a study of nine platforms in Saudi adults found that daily Snapchat use was associated with poorer fruit and vegetable intake but daily LinkedIn and WhatsApp use were associated with a healthier diet (30), and social media exposure has also been used deliberately to increase vegetable consumption (31). These reports may suggest that the relationship between platform use and dietary behavior could depend on the platform, the content encountered, and the food group examined rather than on time spent online alone. Our observations appear to be consistent with this heterogeneity, although its source cannot be identified here, since content exposure was not assessed.

The single association surviving correction—between adherence to the sugary beverages item and lower reported Facebook use—may warrant cautious interpretation despite its adjusted value. Group medians were identical, and the difference appeared to lie in an upper tail of heavier users among non-adherent participants, so it might be better described as a difference in the distribution of heavy use than as a difference in typical use.

Inflammatory and neuroprotective pathways have been proposed as mechanisms linking olive oil intake to mental health, and an olive oil-rich diet has been reported to lower C-reactive protein and interleukin-6 compared with low-fat diets (32). No biological markers were measured in this study, and this background is offered as context for the associations observed rather than as an explanation of them. Similarly, adherence to the Mediterranean diet has been reported to depend on economic circumstances, social connectedness (33), perceived stress (34), physical activity, body weight, and meal timing (35, 36); none of these variables were measured in our sample, and they are summarized here for background purposes only.

## Limitations

Several limitations should be considered. The sample was small, predominantly female (73.0%), young (56.5% were aged 18 to 25), highly educated (81.7% held a university degree or higher), and recruited through the authors’ networks, which may introduce selection bias and could limit generalizability to the wider adult population. Adjustment was possible only for sex, age band, and educational level, and so residual confounding by unmeasured lifestyle, socioeconomic, and clinical factors may remain; the adjusted models explained less than 10% of the variance in symptom scores. The cross-sectional design does not allow inferences about direction, and reverse causation is possible, in which anxiety symptoms might shape dietary choices and patterns of social media use rather than the reverse.

Dietary intake and social media use were self-reported. Three adherence items were administered at thresholds specific to this survey, and the composite score was reported as a modified score; sensitivity analyses using an 11-item score restricted to the items administered at the original thresholds returned the same results, and the analyses reported above were conducted at the item level. Social media use was captured using five fixed time categories for Instagram, Facebook, and Twitter/X, censored at both ends of its range and without a zero-minute option; for Facebook and Twitter/X, the lowest category was the modal response, so the distributions for these platforms are concentrated at the lower end of the scale. Video-based platforms were not assessed. The Spanish and Russian instruments were translated from the published English versions by native speakers, and the language of completion was not recorded, so measurement equivalence across versions was not assessed; internal consistency in the pooled sample was acceptable for both subscales. Country or region of residence was not recorded.

Numerous comparisons were performed, so the likelihood of false-positive findings may be elevated; associations that did not survive correction are reported as exploratory. The study should therefore be regarded as hypothesis-generating rather than confirmatory.

## Conclusion

Olive oil intake and restricted red and processed meat intake each appeared to be associated with lower anxiety scores, in the direction prior evidence would predict, and both associations appeared to hold after adjustment for sex, age band, and educational level. The effects are small and did not survive correction for multiple testing, and the study is accordingly reported as hypothesis-generating rather than confirmatory.

One further observation may be of more general interest. Overall adherence, tested as a summary exposure, did not appear to be related to symptom scores or to social media use, whereas several item-level associations did, and these differed across food groups, running in opposite directions for different items. A composite score sums those components as though they acted alike, and in this sample, that summation returned a null result while its constituents did not. This could be a reason to test dietary components individually rather than to rely on total adherence scores as the exposure of interest, and it might apply whether the specific associations reported here replicate.

Olive oil quantity and red and processed meat intake may be priorities for confirmatory testing in adequately powered samples, with both HADS subscales as outcomes.

## Data Availability

The raw data supporting the conclusions of this article will be made available by the authors, upon request.
