# Supplementary material for: Mediterranean diet adherence, social media use, and anxiety symptoms: an exploratory cross-sectional pilot survey
Source: Front Nutr. 2026 Oct 14;13:1883903. doi: 10.3389/fnut.2026.1883903 (PMC13713344; doi:10.3389/fnut.2026.1883903)
Supplement: Supplementary file 1 [file Table_1.docx]

# Supplementary Material S1

## Questionnaire as administered

The survey was self-administered online using Google Forms and was available in English, Spanish and Russian. The items are reproduced below in English, with the response options as presented to participants. The dietary items were adapted from the PREDIMED Mediterranean Diet Adherence Screener (MEDAS); three were administered at thresholds specific to this survey and are marked with an asterisk.

### Section 1. Demographic items

1. Sex

Response options: Female; Male

2. Age

Response options: 18–25; 26–35; 36–45; 46–55; 56–65

3. Education

Response options: Secondary studies; Vocational training module; University degree or higher

### Section 2. Dietary items (14-item modified Mediterranean Diet Score, mMEDS)

Each item was answered Yes or No and scored 1 for adherence and 0 for non-adherence, giving a total score range of 0 to 14. No item was reverse-coded. Three items were administered at thresholds specific to this survey and are marked with an asterisk: vegetable intake at four or more servings daily, red and processed meat at fewer than two servings per week, and wine at more than seven glasses per week.

4. Is olive oil the main source of oil when cooking?
5. Does it consumedaily 4 tablespoons (60 ml)or more olive oil? This includes oil used for frying, salads, meals eaten out...
6. Do you use homemade sauces to accompany pasta, rice...twice or more a week? (sauces made from olive oil, onion, tomato, garlic...)
7. Does it consume4 or more servings dailyof vegetables? *
8. Does it consume3 or more servings dailyof fruit? (1 portion = 1 fruit or 120g)
9. Do you eat nuts?3 or more times a week?
10. Does it consume3 servings or morefishper week? (1 portion=100-150g)
11. Does it consume3 servings or moreof legumesper week(1 portion = approximately 150g)
12. Do you eat chicken or turkey more often than beef or pork? (Include hamburgers and sausages)
13. Does it consumeless than 2 servingsof red or processed meatper week(1 portion = 75g) *
14. Does it consumedaily less than 1 tablespoon (15ml)) of butter, margarine or cream?
15. Does it consume2 servings or lessof processed snacksper week? (snacks being cookies, donuts, cakes...)
16. Does it consumeless than 1can of sugary sodaper day?
17. Does it consumemore than 7wine glassesper week? *

Response options for all items in this section: Yes; No.

### Section 3. Social media items

18. Do you use social media?

Response options: Yes; No.

19. How many minutes a day do you spend on Instagram?

Response options: 10 minutes or less; 30 minutes; 50 minutes; 70 minutes; 90 minutes or more.

20. How many minutes a day do you spend connected to Facebook?

Response options: 10 minutes or less; 30 minutes; 50 minutes; 70 minutes; 90 minutes or more.

21. How many minutes a day do you spend connected to Twitter/X?

Response options: 10 minutes or less; 30 minutes; 50 minutes; 70 minutes; 90 minutes or more.

22. Where do you usually connect to the internet from?

### Section 4. Hospital Anxiety and Depression Scale (HADS)

The HADS was administered in its published English version, comprising seven anxiety items (odd-numbered) and seven depression items (even-numbered), each scored 0 to 3 and each subscale ranging from 0 to 21. The instrument is under copyright and is therefore not reproduced here; it is available from the copyright holder under licence. The original description is: Zigmond AS, Snaith RP. The Hospital Anxiety and Depression Scale. Acta Psychiatr Scand 1983;67(6):361–370.

Responses collected in Spanish and Russian were harmonised to a single English label set before scoring. The mapping from each response label to its 0–3 score is implemented in Supplementary Material S3.

### Scoring procedure

mMEDS: one point per item met, summed across the fourteen dietary items (range 0–14). HADS anxiety: sum of the seven odd-numbered items (range 0–21). HADS depression: sum of the seven even-numbered items (range 0–21). HADS scores were classified as normal (0–7), borderline (8–10) and abnormal (11–21). Social media responses were assigned the values 10, 30, 50, 70 and 90 minutes for analysis.
