# Supplementary material for: Mediterranean diet adherence, social media use, and anxiety symptoms: an exploratory cross-sectional pilot survey
Source: Front Nutr. 2026 Oct 14;13:1883903. doi: 10.3389/fnut.2026.1883903 (PMC13713344; doi:10.3389/fnut.2026.1883903)
Supplement: Supplementary file 2 [file Table_2.docx]

# Supplementary Material S2

## STROBE Statement — checklist for cross-sectional studies

Manuscript ID 1883903. Locations refer to sections of the revised manuscript.

| **Section** | **Item** | **Recommendation** | **Reported in** |
| --- | --- | --- | --- |
| Title and abstract | 1a | Indicate the study design with a commonly used term in the title or the abstract | Title |
| Title and abstract | 1b | Provide in the abstract an informative and balanced summary of what was done and what was found | Abstract |
| Introduction | 2 | Explain the scientific background and rationale for the investigation being reported | Introduction |
| Introduction | 3 | State specific objectives, including any prespecified hypotheses | Methods, Hypotheses |
| Methods | 4 | Present key elements of study design early in the paper | Methods, Study design and participants |
| Methods | 5 | Describe the setting, locations, and relevant dates, including periods of recruitment, exposure, follow-up, and data collection | Methods, Study design and participants |
| Methods | 6a | Give the eligibility criteria, and the sources and methods of selection of participants | Methods, Study design and participants |
| Methods | 7 | Clearly define all outcomes, exposures, predictors, potential confounders, and effect modifiers | Methods, Dietary/Psychological/Social media assessment |
| Methods | 8 | For each variable of interest, give sources of data and details of methods of assessment | Methods, Dietary/Psychological/Social media assessment; Supplementary S1 |
| Methods | 9 | Describe any efforts to address potential sources of bias | Methods, Statistical analysis; Limitations |
| Methods | 10 | Explain how the study size was arrived at | Methods, Study design and participants |
| Methods | 11 | Explain how quantitative variables were handled in the analyses | Methods, Statistical analysis |
| Methods | 12a | Describe all statistical methods, including those used to control for confounding | Methods, Statistical analysis |
| Methods | 12b | Describe any methods used to examine subgroups and interactions | Methods, Statistical analysis; Supplementary Figures S2–S3 |
| Methods | 12c | Explain how missing data were addressed | Methods, Study design and participants |
| Methods | 12d | If applicable, describe analytical methods taking account of sampling strategy | Not applicable |
| Methods | 12e | Describe any sensitivity analyses | Methods, Dietary assessment; Results, Primary comparisons |
| Results | 13a | Report numbers of individuals at each stage of the study | Results, Sample characteristics; Figure 1 |
| Results | 13b | Give reasons for non-participation at each stage | Methods, Study design and participants |
| Results | 13c | Consider use of a flow diagram | Figure 1 |
| Results | 14a | Give characteristics of study participants and information on exposures and potential confounders | Results, Sample characteristics; Table 1 |
| Results | 14b | Indicate number of participants with missing data for each variable of interest | Methods, Study design and participants; Tables 4 and 5 |
| Results | 15 | Report numbers of outcome events or summary measures | Results; Tables 1, 4 and 5 |
| Results | 16a | Give unadjusted estimates and, if applicable, confounder-adjusted estimates and their precision | Results, Primary comparisons; Tables 4 and 5 |
| Results | 16b | Report category boundaries when continuous variables were categorized | Methods, Psychological and Social media assessment |
| Results | 17 | Report other analyses done, such as analyses of subgroups and interactions, and sensitivity analyses | Results; Supplementary Figures S2–S3 |
| Discussion | 18 | Summarise key results with reference to study objectives | Discussion |
| Discussion | 19 | Discuss limitations, taking into account sources of potential bias or imprecision | Discussion, Limitations |
| Discussion | 20 | Give a cautious overall interpretation of results | Discussion; Conclusion |
| Discussion | 21 | Discuss the generalisability of the study results | Discussion, Limitations |
| Other information | 22 | Give the source of funding and the role of the funders | Funding |
